# Metabolic profiling–multitude of technologies with great research potential, but (when) will translation emerge?

**DOI:** 10.1093/ije/dyw305

**Published:** 2016-10-27

**Authors:** Mika Ala-Korpela, George Davey Smith

**Affiliations:** ^1^Computational Medicine, Faculty of Medicine, University of Oulu and Biocenter Oulu, Oulu, Finland; ^2^Medical Research Council Integrative Epidemiology Unit and School of Social and Community Medicine, University of Bristol, Bristol, UK

Metabolic phenotyping, nowadays most often termed ‘metabolomics’, is becoming increasingly applied in molecular epidemiology, particularly in concert with genomics. Since Jeremy Nicholson and colleagues coined the term ‘metabonomics’ in 1999,^1^ over 15 000 publications have appeared under this conceptual and technological umbrella (a Pubmed search on 15 August 2016 at [http://www.ncbi.nlm.nih.gov/pubmed/] with metabonomics or metabolomics or lipidomics). Most of the published works have been, and still continue to be, methodologically oriented^2^ and thereby bear little direct relevance to applied epidemiology. Particularly the spectroscopy-based chemometric approaches–typically aiming at classification of individuals with or without a particular disease–have for a long time (mis)guided metabolomics research.^3–9^ Many of the limitations of these types of multivariate metabolomics applications are currently well understood: overtraining of classification models with high numbers of variables (typically spectral data points), cross-sectional study settings with very small numbers of individuals and no independent replication.^5^^,^^7^^,^^8^ However, some lack of clarity still remains, partly related to some misplaced conceptions as to the scope of truly personalized medicine.^10–13^ Individual diagnostics of polygenic diseases, when both the disease liability^14^ and the metabolic phenotypes^15–17^ are continuous, fundamentally preclude diagnostic models that would provide both high sensitivity and high specificity.^4^^,^^5^^,^^7^^,^^18–20^ For example, conditions like autism, long considered rigid disease classifications, clearly involve a somewhat arbitrary division of a continuously distributed underlying liability,^21^ limiting attempts at improved binary classification. In addition, many metabolomics applications have ignored confounding in data analyses and interpretations, though it is well established in observational epidemiology that confounding–by lifestyle and socioeconomic factors, or by baseline health status, treatment and medication effects–is prone to affect many associations.^22^^,^^23^

## Getting quantitative and molecular

Recent technological developments resulting in increased numbers of quantitative molecular applications of metabolomics triggered the idea for this themed issue in metabolic phenotyping in epidemiology. The pivotal role of absolute quantification of identified molecular entities in epidemiology and genetics is evident from a multitude of recent applications.^6^^,^^24–28^ The data analysis protocols in mass spectrometry (MS) often build on a quantitative logic, i.e. identification, assignment and evaluation of specific molecular signals. Though most of the MS-based studies have been small or moderate in numbers from the epidemiological perspective, multiple interesting studies have recently been published,^24^^,^^26^^,^^29–31^ including comparisons between two common commercial MS platforms.^32^ On the other hand, a large number of the applications of nuclear magnetic resonance (NMR) spectroscopy in metabolomics have been spectroscopy-based chemometric approaches.^5^^,^^33^ Multiple highly implausible conclusions, for example in relation to coronary heart disease and cancer diagnostics, have been published.^3^^,^^7^ However, during the past 20 years NMR-based lipoprotein subclass profiling has been commercialized and become visible in epidemiology and clinical applications.^34^^,^^35^ NMR spectroscopy can also be used as a general method to quantify multiple molecular constituents in serum and in other biofluids.^6^^,^^28^^,^^36–38^ However, few applications apart from focused lipoprotein profiling,^35^^,^^39^ have been published in epidemiological contexts: only one quantitative serum metabolomics platform being systematically applied in metabolic profiling studies of more than a thousand people.^6^^,^^28^^,^^40^ The pros and cons of NMR and MS for metabolic profiling have been extensively covered in multiple reviews.^2^^,^^6^^,^^41–44^ MS-based lipidomics in epidemiology is reviewed in this issue by Meikle and co-workers,^45^ with the anticipation that further progress is in sight from large, well-characterized cohorts. In addition, a compelling reminder of the current possibilities of *in vivo* metabolic phenotyping, and a vision of how to reach large-scale neurochemical profiling in epidemiological research, is provided in this issue by McKay and Tkáč.^46^

## Combining MS and NMR and notes on replication and causality

So far only a few epidemiological studies have combined MS and NMR methodologies. These are important for increasing the number of metabolic measures studied and also to validate biomarker findings by different technologies. Recently Wahl and colleagues^47^ combined over 400 quantitative measures from serum MS and NMR platforms for over 1600 participants, when studying a multi-omic signature of body weight change in a population-based cohort. Würtz and colleagues^15^ corroborated NMR-based cardiovascular biomarker associations with MS in two population cohorts with 671 and 2289 individuals. In this issue, Vogt and coauthors^48^ characterize associations between serum 25-hydroxyvitamin D concentrations and those of 415 metabolite and lipid measures, quantified by both NMR- and MS-based platforms in 1726 people from a population-based study, KORA F4. Importantly, they also replicated the majority of their findings in an independent population-based study with 6759 individuals for the NMR-based measures and 609 for the MS-based measures. In another study in this issue, Nelson and co-workers^49^ also characterize associations between serum 25-hydroxyvitamin D and serum metabolites, using primarily a non-targeted MS-based approach with eventually 940 compounds identified in eight mutually exclusive chemical classes; lipids, amino acids, xenobiotics, peptides, co-factors and vitamins, carbohydrates, energy metabolites and nucleotides. This is a remarkable metabolic coverage from a technical point of view. Nevertheless, the results are weakened by the lack of replication. Comparison of the results from Nelson and co-workers^49^ with those of Vogt and coauthors^48^ illustrates the importance of biologically independent data; for example, the association of 3-carboxy-4-methyl-5-propyl-2-furanpropanoic acid (CMPF) with 25-hydroxyvitamin D is similarly positive and strong in all three cohorts; however, eicosapentaenoate (EPA) and docosahexaenoate (DHA), which show strong positive associations in the ATBC study,^49^ do not associate with 25-hydroxyvitamin D in KORA F4.^48^ Possible explanation for this discrepancy is the finding in the ATBC study that retinol influenced the associations between 25-hydroxyvitamin D and EPA as well as DHA.^49^

There are of course many possible confounding factors that complicate interpretations of cross-sectional epidemiological studies, particularly when aiming to infer causality,^22^ which Mendelian randomization analyses can attempt to circumvent. A recently published study, applying the Mendelian randomization framework^50–53^ to investigate potential causality in the association between 25-hydroxyvitamin D and schizophrenia, gives an exemplar of how, in certain cases, potential confounding by lifestyle factors can be minimized.^54^ The study findings suggested that associations between schizophrenia and 25-hydroxyvitamin D may not be causal, and, therefore the evidential basis for vitamin D supplementation as a candidate approach for preventing schizophrenia is weakened. Similarly, a recent extensive Mendelian randomization study provides no support for a causal role for 25-hydroxyvitamin D in the risk of coronary artery disease.^55^ On the other hand, in this issue Ong and co-workers^56^ found that genetically lowered 25-hydroxyvitamin D levels were associated with higher susceptibility to ovarian cancer. Along the same lines, genetically lowered 25-hydroxyvitamin D levels have been found to be associated with increased susceptibility to multiple sclerosis.^57^ Thus, in relation to these outcomes, vitamin D sufficiency may be important in delaying onset of or preventing the disease and might thus merit further investigations in long-term randomized controlled trials. These recent applications of the Mendelian randomization framework are interesting examples of causal epidemiology and a welcome reminder that observational association studies, such as those regarding 25-hydroxyvitamin D and circulating lipids and metabolites in this issue,^48^^,^^49^ are often prone to confounding and reverse causation.^22^ Furthermore, an important note here is that replication of observational associations does not mean causation, as previously illustrated in the case of sex hormone-binding globulin and systemic metabolism.^58^ In relation to these key challenges, also in this issue, Swerdlow and co-workers^59^ present important guidance on how to plan and interpret Mendelian randomization studies on disease biomarkers: very relevant in the current era of a wealth of genetic data from genome-wide studies and the increasing number of quantitative metabolomics studies in epidemiology.

The use of Mendelian randomization analysis or randomized controlled trials to assess causality calls for certain prerequisites to be feasible.^53^^,^^60^^,^^61^ One interesting example in this issue is the study by Wang and co-workers^62^ on the effects of hormonal contraception on systemic metabolism. The difficulties in randomizing women to use hormonal contraception or placebo, and randomizing to hormonal or non-hormonal contraception, are obvious. Use of Mendelian randomization analysis to assess the causal effects of oestrogen and progestin is currently limited by the lack of genetic variants that are reliable and consistent proxies for oestrogen and progesterone exposure. Furthermore, even were such variants to be identified, they might not mimic the effects of taking exogenous hormones. To address these difficulties and strengthen causal inference Wang *et al.*^62^ integrated findings from cross-sectional and longitudinal study settings. They assessed a comprehensive molecular profile of 75 metabolic measures and 37 cytokines in up to 5841 women within three population-based cohorts. Women using combined oral contraceptive pills (COCPs) were compared with those who did not use hormonal contraception. Metabolomics profiles were also reassessed for 869 women after 6 years to uncover the metabolic effects of starting, stopping and persistently using hormonal contraception. The extensive metabolic measurements allowed multiple novel findings on the systemic effects of COCPs. Perhaps of greater importance to public health, persistent use of COCPs did not appear to produce cumulative effects over time and the metabolic perturbations were reversed upon discontinuation.^62^

The study by Wang and co-workers^62^ applied a quantitative serum NMR metabolomics platform; one that has commonly been used in epidemiology and genetics.^6^^,^^40^ The platform provides some 150 primary concentration measures. These include a fine-grained lipoprotein subclass profiling, and quantification of circulating fatty acids, amino acids, gluconeogenesis-related metabolites and many other molecules from multiple metabolic pathways, in addition to multiple biomarkers already routinely used in epidemiology.^15–17^^,^^27^^,^^28^^,^^63–67^ The primary measures can also be used to calculate many derived measures and metabolic ratios with potential biological importance. In addition to the study by Wang and co-workers,^62^ three other papers in this issue also apply this methodology, namely the above-mentioned study on serum 25-hydroxyvitamin D by Vogt and co-authors^48^ and two by Würtz and co-workers which look at metabolic profiling of alcohol consumption^68^ and metabolic signatures of birthweight in adulthood.^69^

Alcohol consumption in nearly 10 000 young adults (in a cross-sectional setting in three population-based cohorts) was associated with a complex metabolic signature, comprising both favourable and adverse effects in relation to the risk of cardiovascular disease and type 2 diabetes.^68^ As in the study by Wang and co-workers,^62^ Würtz *et al.*^68^ also complemented the cross-sectional study setting with a longitudinal set-up to be able to better assess the potential causality of the associations. In fact, the metabolic changes associated with the changes in alcohol intake (during 6-year follow-up in 1466 individuals) matched well with the cross-sectional results, increasing evidence that these changes were, at least partly, due to alcohol consumption. The results of Würtz *et al.*^68^ can be interpreted along the lines of recent extensive Mendelian randomization analyses,^70^ suggesting that reduction of alcohol consumption, even for light to moderate drinkers, is beneficial for cardiovascular health, with the obvious implication that Mendelian randomization studies of the influence of alcohol on the metabolome would be a natural extension of this work. The study looking at the metabolic signatures of birthweight in adulthood in 18 288 people is one the largest epidemiological metabolomics studies published to date.^69^ These data show that lower birthweight is adversely associated with a wide range of established and emerging circulating cardiometabolic biomarkers in adulthood. However, the magnitudes of metabolic aberrations were weak (although statistically significant) and the authors questioned their public health relevance.^69^ The pattern of metabolic deviations associated with lower birthweight resembled the metabolic signature of higher adult body mass index (R^2^ 0.77) with 1 kg lower birthweight being associated with similar metabolic aberrations as caused by 0.92 units higher body mass index in adulthood. The authors suggested that shared underlying metabolic pathways may be involved and concluded that birthweight is only a weak indicator of metabolic risk in adulthood.^69^

## Quantitative molecular data–the base for a multitude of statistical and clinical appoaches

When metabolomics gets quantitative,^5^^,^^6^ it no longer matters if the technology is based on MS or NMR (or whatever methodology), the output just becomes a list of molecular concentrations, the length and details of which depend on the method in question. This marks a fundamental distinction from diagnostics-oriented spectroscopy-based chemometric approaches; moving from (potentially thousands of) spectral data points to (typically up to a few hundred) identified molecular measures dramatically simplifies the statistical analyses and interpretations of epidemiological data. Of course, biological and clinical appreciation would need to be integrated into the metabolomics study rationale to change the search for non-existent binary disease states in the case of polygenic outcomes.^5^^,^^6^^,^^9^ Although the transformation from spectra to quantitative molecular measures is a challenge in itself, it is not discussed here since the topic falls into the domain of analytical chemistry and a plentiful literature is available.^6^^,^^34^^,^^36^^,^^42^^,^^71–75^ Nevertheless from the epidemiology perspective, with quantitative metabolomics data, i.e. with that list of molecular concentrations, all is back to basics and business as usual, apart from the fact that the list of molecular variables is longer than it has usually been in epidemiological studies. All standard statistical approaches can be applied in a straightforward manner, including adjustments for potential confounding factors.^6^^,^^22^^,^^24^^,^^25^^,^^28^ Of course, as with any quantitative molecular data, the analyses are by no means limited to standard approaches but, for example, multivariate non-linear approaches,^76^^,^^77^ network analyses,^77^^,^^78^ pathway approaches^79^^,^^80^ and integration of multi-omic data^26^^,^^47^^,^^75^^,^^81–86^ are all feasible. In fact, this is in contrast to spectral-based approaches in which these types of analyses, aiming for detailed biological understanding, would mostly be impossible to perform and interpret at the molecular level. Fearnley and Inouye,^87^ in their review in this issue, survey epidemiological studies that leverage metabolomics and multi-omics to gain insight into disease mechanisms. Whereas they emphasize the role of quantitative metabolic data in biomarker identification and in understanding the metabolic underpinnings of diseases, they also underline limitations and discuss potential solutions in relation to statistical power issues with respect to sample sizes and limited coverage of relevant metabolites. They advocate a conceptual shift from metabolite concentrations towards experiments and graph-theoretical analyses based on the reactions themselves, and envision the identification of subgroups of individuals enriched for variation in relevant subregions of a reaction network in population-level epidemiological studies.^87^

In another review in this issue, Sattar and colleagues discuss the applications and use of metabolomics in cardiometabolic intervention studies and trials.^88^ They express their concerns regarding the small scale and focus on surrogate outcomes in most metabolomics studies in the area. Advancing a list of recommendations for future biomarker studies, they call for multi-expertise research coalitions to work together for rigorous experimental study designs, with an early focus on truly relevant clinical questions:^88^ the latter an issue recently elaborated in relation to clinical research in general by Ioannidis.^89^ They also present an interesting and critical discussion on the potential role of metabolomics in predicting drug responses, and elaborate this in the case of statins. Their points are well made from the clinical point of view and very valuable to consider. Generally, the idea of individual metabolic phenotypes is alluring^90–92^ and might in some cases provide additional and predictive value, for not only drugs but dietary substances as well.^41^^,^^93^ However this does not necessarily translate into clinical relevance or applicability and, as the review by Sattar and colleagues^88^ indicates, applications of metabolomics in clinical trials are scarce. Recently Würtz and co-workers published a proof-of-concept study^17^–a, “natural”, clinical trial of statin effects^94^–in which they overcame the lack of metabolomics data in randomized controlled trials by using serially collected blood samples in population-based cohorts, in which a subset of individuals had started to use statins during follow-up. To verify that the observed metabolic changes were actually due to the effects of statins, the analyses were corroborated via Mendelian randomization analyses using a genetic variant in the *HMGCR* gene as an unconfounded proxy for the pharmacological action of statins. In fact, this type of combination of metabolomics data with genetic data in a large number of individuals readily extends to studies of all drugs with established genetic proxies mimicking their pharmacological action. With increasing numbers of extensive metabolomics and genetic data becoming available, we anticipate that comprehensive metabolic profiles of drug targets are likely to augment drug development in preclinical stages. Applications of two-sample Mendelian randomization would allow the gene-risk factor and gene-outcome associations to be taken from different data sources.^95^

An early example in drug research is the Consortium for Metabonomic Toxicology, a collaboration that involved several pharmaceutical companies in applications of metabolomics to preclinical drug safety studies; this consortium, via the measurement of a dataset of NMR spectra of rodent urine and serum samples, built a predictive system for liver and kidney toxicity.^96^ However, these approaches have not been widely adopted by the pharmaceutical industry. This reluctance was likely due to the fact that the methodologies originally used were spectroscopy-based and chemometrics-driven and thereby not sufficiently sensitive, quantitative, molecular-specific or platform-independent to permit routine or widespread implementation.^97^ With the recent shift and developments in metabolomics towards quantitative molecular methodologies,^6^^,^^32^ it may well be worthwhile to revisit this approach. In biomedical omics applications in general, it is also worth noting that there have been intensive developments regarding proteomics methods during the past few years, and their quantitative applications are likely soon to become feasible in large-scale studies to complement the information from other omics domains.^98–100^

## Commercial products and peer-reviewed publications

Intellectual property rights for most of the quantitative metabolomics platforms, particularly those applied in most epidemiological and clinical applications to date, are owned by companies.^6^^,^^32^ This is not surprising as such, since innovations are, manifestly, often developed by such enterprises. Ioannidis has recently reflected upon biomedical innovations and scientific peer review.^89^^,^^101^ He makes the point that ’stealth research’ is prone to create ambiguity about what evidence can be trusted in a mix of (possibly) ground-breaking ideas, aggressive corporate announcements and mass media hype.^89^ Even more recently, he has called for scientific peer-reviewed articles as a requirement for technologies aiming to affect health care at large.^101^ In this sense–of publishing peer-reviewed scientific articles–the current key metabolomics methodologies in epidemiology are reasonably represented.^6^^,^^32^ In fact, it appears that many companies operating in the metabolomics arena have made scientific peer-reviewed articles part of their business strategy. This is logical in light of the great potential for commercial benefit provided by independent technological validation by the scientific community. This is something that cannot be achieved by patents or intellectual property rights alone; high-quality peer-reviewed publications are hard to copy or buy. However what still partly remains, even in the peer-reviewed scientific literature, is ungrounded hype and expectations of omics sciences transforming not only biological knowledge, but also medicine and public health.^102^ In relation to clinical applications, we should also keep in mind the problematic pathway from the announcement of new biomarkers to their integration into predictive and diagnostic models or validated target identification.^18^^,^^20^^,^^103–107^

## Is the future for metabolic phenotyping in epidemiology precarious?

In the history of science and medicine, remarkable leaps in progress have often been made due to novel physical or chemical technologies. The new omics methodologies have already taken a big step, particularly in combination with genomic data, but also generally in biomedical sciences. Quantitative molecular technologies have been developed, many of which are ready for integration into large-scale epidemiological studies. There is much interest in ‘multiomic’ science, in addition to epidemiological and potential clinical applications. However, considerable hype has also been generated, often in connection with the concept of personalized or precision medicine and clinical diagnostic applications. We suggest that for the future meaningful development of metabolic phenotyping in epidemiological and (potentially) clinical settings, much of the early metabolomics literature should be put behind us, just as we needed to discard a large (and largely meaningless) ‘candidate gene’ literature with the arrival of genome-wide association studies.^108^ We should embrace some of the basic requirements of good molecular epidemiology: molecular identification and quantification, large-scale studies, independent biological replication of results, and an appreciation of confounding with the aim of understanding causation. We should combine different metabolomics methodologies and multiomics combinations to triangulate findings, explore multiple angles and put findings into a biological perspective. Furthermore we should apply appropriate statistical tools (noting caveats related to multiple testing) and, maybe most importantly, apply self-critical interpretation of statistical results, biological implications and potential clinical applicability. Last, we should carefully reflect–against all the hopes and hypes of personalized medicine–on the fundamentals of biological processes in polygenic conditions, and the epidemiological connotations of this with respect to the articulation of population and individual perspectives.^10^^,^^11^ As demonstrated by many excellent contributions in this themed issue, large-scale metabolic phenotyping, together with many other omics technologies, are already here to enrich epidemiology and eventually to make irreversible headway in the era of systems epidemiology. 

## Funding

MAK has received funding from the Sigrid Juselius Foundation and strategic research funding from the University of Oulu, Finland. MAK and GDS work in a Unit that receives funds from the University of Bristol and UK Medical Research Council (MC_UU_12013/1). The funders had no role in study design, decision to publish, or preparation of the manuscript.

## References

[1] Nicholson JK Lindon JC Holmes E. ‘Metabonomics’: understanding the metabolic responses of living systems to pathophysiological stimuli via multivariate statistical analysis of biological NMR spectroscopic data. Xenobiotica 1999;29:1181–89. 10598751 10.1080/004982599238047

[2] Dunn WB Broadhurst DI Atherton HJ Goodacre R Griffin JL. Systems level studies of mammalian metabolomes: the roles of mass spectrometry and nuclear magnetic resonance spectroscopy. Chem Soc Rev 2011;40:387–426. 20717559 10.1039/b906712b

[3] Ala-Korpela M. Potential role of body fluid ^1^H NMR metabonomics as a prognostic and diagnostic tool. Expert Rev Mol Diagn 2007;7:761–73. 18020906 10.1586/14737159.7.6.761

[4] Ala-Korpela M. Critical evaluation of ^1^H NMR metabonomics of serum as a methodology for disease risk assessment and diagnostics. Clin Chem Lab Med 2008;46:27–42. 18020967 10.1515/CCLM.2008.006

[5] Ala-Korpela M Kangas AJ Soininen P. Quantitative high-throughput metabolomics: a new era in epidemiology and genetics. Genome Med 2012;4:36. 22546737 10.1186/gm335 PMC3446264

[6] Soininen P Kangas AJ Würtz P Suna T Ala-Korpela M. Quantitative serum nuclear magnetic resonance metabolomics in cardiovascular epidemiology and genetics. Circ Cardiovasc Genet 2015;8:192–206. 25691689 10.1161/CIRCGENETICS.114.000216

[7] Ala-Korpela M. Metabolomics in cardiovascular medicine: Not personalised, not diagnostic. Eur J Prev Cardiol 2016 [epub ahead of print]. doi: 10.1177/2047487316664443. 10.1177/2047487316664443 27514945

[8] Xia J Broadhurst DI Wilson M Wishart DS. Translational biomarker discovery in clinical metabolomics: an introductory tutorial. Metabolomics 2013;9:280–99. 23543913 10.1007/s11306-012-0482-9 PMC3608878

[9] Madsen R Lundstedt T Trygg J. Chemometrics in metabolomics - a review in human disease diagnosis. Anal Chim Acta 2010;659:23–33. 20103103 10.1016/j.aca.2009.11.042

[10] Rose G. Sick individuals and sick populations. Int J Epidemiol 1985;14:32–38. 3872850 10.1093/ije/14.1.32

[11] Davey Smith G. Epidemiology, epigenetics and the ‘Gloomy Prospect’: embracing randomness in population health research and practice. Int J Epidemiol 2011;40:537–62. 21807641 10.1093/ije/dyr117

[12] Hey SP Kesselheim AS. Countering imprecision in precision medicine. Science 2016;353:448–49. 27471295 10.1126/science.aaf5101

[13] Davey Smith G Relton CL Brennan P. Chance, choice and cause in cancer aetiology: individual and population perspectives. Int J Epidemiol 2016;45:605-13. 27565178 10.1093/ije/dyw224

[14] Plomin R Haworth CMA Davis OSP. Common disorders are quantitative traits. Nat Rev Genet 2009;10:872–78. 19859063 10.1038/nrg2670

[15] Würtz P Havulinna AS Soininen P Metabolite profiling and cardiovascular event risk: a prospective study of 3 population-based cohorts. Circulation 2015;131:774–85. 25573147 10.1161/CIRCULATIONAHA.114.013116 PMC4351161

[16] Würtz P Mäkinen V-P Soininen P Metabolic signatures of insulin resistance in 7,098 young adults. Diabetes 2012;61:1372–80. 22511205 10.2337/db11-1355 PMC3357275

[17] Würtz P Wang Q Soininen P Metabolomic profiling of statin use and genetic inhibition of HMG-CoA reductase. J Am Coll Cardiol 2016;67:1200–10. 26965542 10.1016/j.jacc.2015.12.060 PMC4783625

[18] Ware JH. The limitations of risk factors as prognostic tools. N Engl J Med 2006;355:2615–17. 17182986 10.1056/NEJMp068249

[19] Wang TJ. Assessing the role of circulating, genetic, and imaging biomarkers in cardiovascular risk prediction. Circulation 2011;123:551–65. 21300963 10.1161/CIRCULATIONAHA.109.912568 PMC3059807

[20] Niiranen TJ Vasan RS. Epidemiology of cardiovascular disease: recent novel outlooks on risk factors and clinical approaches. Exp Rev Cardiov Ther 2016;14:855–69. 10.1080/14779072.2016.1176528 PMC4961207 27057779

[21] Robinson EB Pourcain BS Anttila V Genetic risk for autism spectrum disorders and neuropsychiatric variation in the general population. Nat Genet 2016;48:552–55. 26998691 10.1038/ng.3529 PMC4986048

[22] Davey Smith G Lawlor DA Harbord R Timpson N Day I Ebrahim S. Clustered environments and randomized genes: a fundamental distinction between conventional and genetic epidemiology. PLoS Med 2007;4:e352. 18076282 10.1371/journal.pmed.0040352 PMC2121108

[23] Baig F Pechlaner R Mayr M. Caveats of untargeted metabolomics for biomarker discovery. J Am Coll Cardiol 2016;68:1294–96. 27634120 10.1016/j.jacc.2016.05.098

[24] Suhre K Shin S-Y Petersen A-K Human metabolic individuality in biomedical and pharmaceutical research. Nature 2011;477:54–60. 21886157 10.1038/nature10354 PMC3832838

[25] Suhre K Wallaschofski H Raffler J A genome-wide association study of metabolic traits in human urine. Nat Genet 2011;43:565–69. 21572414 10.1038/ng.837

[26] Suhre K Raffler J Kastenmüller G. Biochemical insights from population studies with genetics and metabolomics. Arch Biochem Biophys 2016;589:168-76. 26432701 10.1016/j.abb.2015.09.023

[27] Kettunen J Tukiainen T Sarin A-P Genome-wide association study identifies multiple loci influencing human serum metabolite levels. Nat Genet 2012;44:269–76. 22286219 10.1038/ng.1073 PMC3605033

[28] Kettunen J Demirkan A Würtz P Genome-wide study for circulating metabolites identifies 62 loci and reveals novel systemic effects of LPA. Nat Commun 2016;7:11122. 27005778 10.1038/ncomms11122 PMC4814583

[29] Shin S-Y Fauman EB Petersen A-K An atlas of genetic influences on human blood metabolites. Nat Genet 2014;46:543–50. 24816252 10.1038/ng.2982 PMC4064254

[30] Menni C Kastenmüller G Petersen A-K Metabolomic markers reveal novel pathways of ageing and early development in human populations. Int J Epidemiol 2013;42:1111–19. 23838602 10.1093/ije/dyt094 PMC3781000

[31] Wang TJ Larson MG Vasan RS Metabolite profiles and the risk of developing diabetes. Nat Med 2011;17:448–53. 21423183 10.1038/nm.2307 PMC3126616

[32] Yet I Menni C Shin S-Y Genetic influences on metabolite levels: a comparison across metabolomic platforms. PLoS One 2016;11:e0153672. 27073872 10.1371/journal.pone.0153672 PMC4830611

[33] Dona AC Coffey S Figtree G. Translational and emerging clinical applications of metabolomics in cardiovascular disease diagnosis and treatment. Eur J Prev Cardiol 2016 [epub ahead of print]. doi: 10.1177/2047487316645469. 10.1177/2047487316645469 27103630

[34] Ala-Korpela M. ^1^H NMR spectroscopy of human blood plasma. Prog Nucl Magn Reson Spectrosc 1995;27:475–554.

[35] Mallol R Rodriguez MA Brezmes J Masana L Correig X. Human serum/plasma lipoprotein analysis by NMR: application to the study of diabetic dyslipidemia. Prog Nucl Magn Reson Spectrosc 2013;70:1–24. 23540574 10.1016/j.pnmrs.2012.09.001

[36] Mierisová S Ala-Korpela M. MR spectroscopy quantitation: a review of frequency domain methods. NMR Biomed 2001;14:247–59. 11410942 10.1002/nbm.697

[37] Dona AC Jimenez B Schäfer H Precision high-throughput proton NMR spectroscopy of human urine, serum and plasma for large-scale metabolic phenotyping. Anal Chem 2014;86:9887-94. 25180432 10.1021/ac5025039

[38] Demirkan A Henneman P Verhoeven A Insight in genome-wide association of metabolite quantitative traits by exome sequence analyses. PLoS Genet 2015;11:e1004835. 25569235 10.1371/journal.pgen.1004835 PMC4287344

[39] Mora S Buring JE Ridker PM. Discordance of low-density lipoprotein (LDL) cholesterol with alternative LDL-related measures and future coronary events. Circulation 2014;129:553–61. 24345402 10.1161/CIRCULATIONAHA.113.005873 PMC4501252

[40] Soininen P Kangas AJ Würtz P High-throughput serum NMR metabonomics for cost-effective holistic studies on systemic metabolism. Analyst 2009;134:1781–85. 19684899 10.1039/b910205a

[41] van Duynhoven JPM Jacobs DM. Assessment of dietary exposure and effect in humans: the role of NMR. Prog Nucl Magn Reson Spectrosc 2016;96:58-72. 27573181 10.1016/j.pnmrs.2016.03.001

[42] Lind MV Savolainen OI Ross AB. The use of mass spectrometry for analysing metabolite biomarkers in epidemiology: methodological and statistical considerations for application to large numbers of biological samples. Eur J Epidemiol 2016;31:717-33. 27230258 10.1007/s10654-016-0166-2

[43] Quehenberger O Dennis EA. The human plasma lipidome. N Engl J Med 2011;365:1812–23. 22070478 10.1056/NEJMra1104901 PMC3412394

[44] Rankin NJ Preiss D Welsh P The emergence of proton nuclear magnetic resonance metabolomics in the cardiovascular arena as viewed from a clinical perspective. Atherosclerosis 2014;237:287–300. 25299963 10.1016/j.atherosclerosis.2014.09.024 PMC4232363

[45] Mundra PA Shaw JE Meikle PJ. Lipidomic analyses in epidemiology. Int J Epidemiol 2016;45:1329–38. 27286762 10.1093/ije/dyw112

[46] McKay J Tkáč I. Quantitative in vivo neurochemical profiling in humans: where are we now? Int J Epidemiol 2016;45: 1339–50. 27794521 10.1093/ije/dyw235 PMC5100624

[47] Wahl S Vogt S Stückler F Multi-omic signature of body weight change: results from a population-based cohort study. BMC Med 2015;13:48. 25857605 10.1186/s12916-015-0282-y PMC4367822

[48] Vogt S Wahl S Kettunen J Characterization of the metabolic profile associated with serum 25-hydroxyvitamin D: a cross-sectional analysis in population-based data. Int J Epidemiol 2016;45:1469–81. 27605587 10.1093/ije/dyw222 PMC5100623

[49] Nelson SM Panagiotou OA Anic GM Metabolomics analysis of serum 25-hydroxy-vitamin D in the Alpha-Tocopherol, Beta-Carotene Cancer Prevention (ATBC) Study. Int J Epidemiol 2016;45:1458–68. 27524818 10.1093/ije/dyw148 PMC5100614

[50] Davey Smith G Ebrahim S. ‘Mendelian randomization’: can genetic epidemiology contribute to understanding environmental determinants of disease? Int J Epidemiol 2003;32**:** 1-22. 12689998 10.1093/ije/dyg070

[51] Davey Smith G Hemani G. Mendelian randomization: genetic anchors for causal inference in epidemiological studies. Hum Mol Genet 2014;23:R89–98. 25064373 10.1093/hmg/ddu328 PMC4170722

[52] Burgess S Timpson NJ Ebrahim S Davey Smith G. Mendelian randomization: where are we now and where are we going? Int J Epidemiol 2015;44:379–88. 26085674 10.1093/ije/dyv108

[53] Haycock PC Burgess S Wade KH Bowden J Relton C Davey Smith G. Best (but oft-forgotten) practices: the design, analysis, and interpretation of Mendelian randomization studies. Am J Clin Nutr 2016;103:965–78. 26961927 10.3945/ajcn.115.118216 PMC4807699

[54] Taylor AE Burgess S Ware JJ Investigating causality in the association between 25(OH)D and schizophrenia. Sci Rep 2016;6:26496. 27215954 10.1038/srep26496 PMC4877705

[55] Manousaki D Mokry LE Ross S Goltzman D Richards JB. Mendelian randomization studies do not support a role for vitamin D in coronary artery disease. Circ Cardiovasc Genet 2016;9:349–56. 27418593 10.1161/CIRCGENETICS.116.001396

[56] Ong JS Cuellar-Partida G Lu Y Association of vitamin D levels and risk of ovarian cancer: a Mendelian randomization study. Int J Epidemiol 2016;45:1619–30. 27594614 10.1093/ije/dyw207 PMC5100621

[57] Mokry LE Ross S Ahmad OS Vitamin D and risk of multiple sclerosis: a Mendelian randomization study. PLoS Med 2015;12:e1001866. 26305103 10.1371/journal.pmed.1001866 PMC4549308

[58] Wang Q Kangas AJ Soininen P Sex hormone-binding globulin associations with circulating lipids and metabolites and the risk for type 2 diabetes: observational and causal effect estimates. Int J Epidemiol 2015;44:623-37. 26050255 10.1093/ije/dyv093

[59] Swerdlow DI Kuchenbaecker KB Shah S Selecting instruments for Mendelian randomization in the wake of genome-wide association studies. Int J Epidemiol 2016;45:1600–16. 27342221 10.1093/ije/dyw088 PMC5100611

[60] Evans DM Davey Smith G. Mendelian randomization: new applications in the coming age of hypothesis-free causality. Annu Rev Genomics Hum Genet 2015;16:327–50. 25939054 10.1146/annurev-genom-090314-050016

[61] Collins R Reith C Emberson J Interpretation of the evidence for the efficacy and safety of statin therapy. Lancet 2016 [epub ahead of print]. doi: 10.1016/S0140-6736(16)31357-5. 10.1016/S0140-6736(16)31357-5 27616593

[62] Wang Q Würtz P Auro K Effects of hormonal contraception on systemic metabolism: cross-sectional and longitudinal evidence. Int J Epidemiol 2016;45:1445–57. 27538888 10.1093/ije/dyw147 PMC5100613

[63] Würtz P Kangas AJ Soininen P Lipoprotein subclass profiling reveals pleiotropy in the genetic variants of lipid risk factors for coronary heart disease: a note on Mendelian randomization studies. J Am Coll Cardiol 2013;62:1906–08. 24055740 10.1016/j.jacc.2013.07.085

[64] Würtz P Wang Q Kangas AJ Metabolic signatures of adiposity in young adults: Mendelian randomization analysis and effects of weight change. PLoS Med 2014;11:e1001765. 25490400 10.1371/journal.pmed.1001765 PMC4260795

[65] Fischer K Kettunen J Würtz P *.* Biomarker profiling by nuclear magnetic resonance spectroscopy for the prediction of all-cause mortality: an observational study of 17,345 persons. PLoS Med 2014;11:e1001606. 24586121 10.1371/journal.pmed.1001606 PMC3934819

[66] Auro K Joensuu A Fischer K A metabolic view on menopause and ageing. Nat Commun 2014;5:4708. 25144627 10.1038/ncomms5708

[67] Kujala UM Mäkinen V-P Heinonen I Long-term leisure-time physical activity and serum metabolome. Circulation 2013;127:340–48. 23258601 10.1161/CIRCULATIONAHA.112.105551

[68] Würtz P Cook S Wang Q Metabolic profiling of alcohol consumption in 9778 young adults. Int J Epidemiol 2016;45:1493–506. 27494945 10.1093/ije/dyw175 PMC5100616

[69] Würtz P Wang Q Niironen M Metabolic signatures of birthweight in 18 288 adolescents and adults. Int J Epidemiol 2016;45:1539–50. 27892411 10.1093/ije/dyw255 PMC5100627

[70] Holmes MV Dale CE Zuccolo L *.* Association between alcohol and cardiovascular disease: Mendelian randomisation analysis based on individual participant data. BMJ 2014;349:g4164. 25011450 10.1136/bmj.g4164 PMC4091648

[71] Mihaleva VV van Schalkwijk DB de Graaf AA A systematic approach to obtain validated partial least square models for predicting lipoprotein subclasses from serum NMR spectra. Anal Chem 2014;86:543–50. 24319989 10.1021/ac402571z

[72] Mihaleva VV Korhonen S-P van Duynhoven J Niemitz M Vervoort J Jacobs DM. Automated quantum mechanical total line shape fitting model for quantitative NMR-based profiling of human serum metabolites. Anal Bioanal Chem 2014;406:3091–102. 24722875 10.1007/s00216-014-7752-5

[73] Alonso A Marsal S Julià A. Analytical methods in untargeted metabolomics: state of the art in 2015. Front Bioeng Biotechnol 2015;3:1514. 10.3389/fbioe.2015.00023 PMC4350445 25798438

[74] Vehtari A Mäkinen V-P Soininen P A novel Bayesian approach to quantify clinical variables and to determine their spectroscopic counterparts in ^1^H NMR metabonomic data. BMC Bioinformatics 2007;8(Suppl 2):S8. 10.1186/1471-2105-8-S2-S8 PMC1892077 17493257

[75] Inouye M Kettunen J Soininen P Metabonomic, transcriptomic, and genomic variation of a population cohort. Mol Syst Biol 2010;6:441. 21179014 10.1038/msb.2010.93 PMC3018170

[76] Mäkinen V-P Soininen P Kangas AJ Triglyceride-cholesterol imbalance across lipoprotein subclasses predicts diabetic kidney disease and mortality in type 1 diabetes: the FinnDiane Study. J Intern Med 2013;273:383–95. 23279644 10.1111/joim.12026

[77] Mäkinen V-P Tynkkynen T Soininen P Metabolic diversity of progressive kidney disease in 325 patients with type 1 diabetes (the FinnDiane Study). J Proteome Res 2012;11:1782–90. 22204613 10.1021/pr201036j

[78] Mäkinen V-P Forsblom C Thorn LM Network of vascular diseases, death and biochemical characteristics in a set of 4,197 patients with type 1 diabetes (the FinnDiane Study). Cardiovasc Diabetol 2009;8:54. 19804653 10.1186/1475-2840-8-54 PMC2763862

[79] Krumsiek J Suhre K Illig T Adamski J Theis FJ. Gaussian graphical modeling reconstructs pathway reactions from high-throughput metabolomics data. BMC Syst Biol 2011;5:21. 21281499 10.1186/1752-0509-5-21 PMC3224437

[80] Krumsiek J Mittelstrass K Do KT Gender-specific pathway differences in the human serum metabolome. Metabolomics 2015;11:1815–33. 26491425 10.1007/s11306-015-0829-0 PMC4605991

[81] Inouye M Ripatti S Kettunen J *.* Novel loci for metabolic networks and multi-tissue expression studies reveal genes for atherosclerosis. PLoS Genet 2012;8:e1002907. 22916037 10.1371/journal.pgen.1002907 PMC3420921

[82] Ala-Korpela M Kangas AJ Inouye M. Genome-wide association studies and systems biology: together at last. Trends Genet 2011;27:493–98. 22018481 10.1016/j.tig.2011.09.002

[83] Ritchie SC Würtz P Nath AP *.* The biomarker GlycA is associated with chronic inflammation and predicts long-term risk of severe infection. Cell Systems 2015;1:293–301. 27136058 10.1016/j.cels.2015.09.007

[84] Petersen A-K Zeilinger S Kastenmüller G Epigenetics meets metabolomics: an epigenome-wide association study with blood serum metabolic traits. Hum Mol Genet 2014;23:534–45. 24014485 10.1093/hmg/ddt430 PMC3869358

[85] Krumsiek J Bartel J Theis FJ. Computational approaches for systems metabolomics. Curr Opin Biotechnol 2016;39:198–206. 27135552 10.1016/j.copbio.2016.04.009

[86] Kastenmüller G Raffler J Gieger C Suhre K. Genetics of human metabolism: an update. Hum Mol Genet 2015;24:R93–R101. 26160913 10.1093/hmg/ddv263 PMC4572003

[87] Fearnley LG Inouye M. Metabolomics in epidemiology: from metabolite concentrations to integrative reaction networks. Int J Epidemiol 2016;45:1319–28. 27118561 10.1093/ije/dyw046 PMC5100607

[88] Rankin NJ Preiss D Welsh P Sattar N. Applying metabolomics to cardiometabolic intervention studies and trials: past experiences and a roadmap for the future. Int J Epidemiol 2016;45:1351–71. 27789671 10.1093/ije/dyw271 PMC5100629

[89] Ioannidis JPA. Why most clinical research is not useful. PLoS Med 2016;13:e1002049. 27328301 10.1371/journal.pmed.1002049 PMC4915619

[90] Clayton TA Lindon JC Cloarec O Pharmaco-metabonomic phenotyping and personalized drug treatment. Nature 2006;440:1073–77. 16625200 10.1038/nature04648

[91] Assfalg M Bertini I Colangiuli D *.* Evidence of different metabolic phenotypes in humans. Proc Natl Acad Sci U S A 2008;105:1420–24. 18230739 10.1073/pnas.0705685105 PMC2234159

[92] Clayton TA Baker D Lindon JC Everett JR Nicholson JK. Pharmacometabonomic identification of a significant host-microbiome metabolic interaction affecting human drug metabolism. Proc Natl Acad Sci U S A 2009;106:14728–33. 19667173 10.1073/pnas.0904489106 PMC2731842

[93] Larmo PS Kangas AJ Soininen P Effects of sea buckthorn and bilberry on serum metabolites differ according to baseline metabolic profiles in overweight women: a randomized crossover trial. Am J Clin Nutr 2013;98:941–51. 23945716 10.3945/ajcn.113.060590 PMC3778864

[94] Voora D Shah SH. Pharmacometabolomics meets genetics. J Am Coll Cardiol 2016;67:1211–13. 26965543 10.1016/j.jacc.2016.01.022

[95] Burgess S Scott RA Timpson NJ Davey Smith G Thompson SG; EPIC-InterAct Consortium. Using published data in Mendelian randomization: a blueprint for efficient identification of causal risk factors. Eur J Epidemiol 2015;30:543–52. 25773750 10.1007/s10654-015-0011-z PMC4516908

[96] Lindon JC Holmes E Nicholson JK. Metabonomics in pharmaceutical RandD. FEBS J 2007;274:1140–51. 17298438 10.1111/j.1742-4658.2007.05673.x

[97] Wishart DS. Emerging applications of metabolomics in drug discovery and precision medicine. Nat Rev Drug Discov 2016;15:473-84. 26965202 10.1038/nrd.2016.32

[98] Hathout Y. Proteomic methods for biomarker discovery and validation. Are we there yet? Exp Rev Proteomics 2015;12:329–31. 10.1586/14789450.2015.1064771 26186709

[99] Williams EG Wu Y Jha P Systems proteomics of liver mitochondria function. Science 2016;352:aad0189. 27284200 10.1126/science.aad0189 PMC10859670

[100] Billing AM Ben Hamidane H Bhagwat AM Complementarity of SOMAscan to LC-MS/MS and RNA-seq for quantitative profiling of human embryonic and mesenchymal stem cells. J Proteomics 2017;150:86–97. 27613379 10.1016/j.jprot.2016.08.023

[101] Ioannidis JPA. Stealth research and Theranos: reflections and update 1 year later. JAMA 2016;316:389–90. 27191700 10.1001/jama.2016.6986

[102] Ioannidis JPA. Expectations, validity, and reality in omics. J Clin Epidemiol 2010;63:945–49. 20573481 10.1016/j.jclinepi.2010.04.002

[103] Rockhill B Kawachi I Colditz GA. Individual risk prediction and population-wide disease prevention. Epidemiol Rev 2000;22:176–80. 10939025 10.1093/oxfordjournals.epirev.a018017

[104] Poste G. Bring on the biomarkers. Nature 2011;469:156–57. 21228852 10.1038/469156a

[105] Ioannidis JPA Tzoulaki I. Minimal and null predictive effects for the most popular blood biomarkers of cardiovascular disease. Circ Res 2012;110:658–62. 22383708 10.1161/RES.0b013e31824da8ad

[106] Moons KGM Altman DG Reitsma JB *.* Transparent Reporting of a multivariable prediction model for Individual Prognosis or Diagnosis (TRIPOD): explanation and elaboration. Ann Intern Med 2015;162:W1–73. 25560730 10.7326/M14-0698

[107] Vargas AJ Harris CC. Biomarker development in the precision medicine era: lung cancer as a case study. Nat Rev Cancer 2016;16:525–37. 27388699 10.1038/nrc.2016.56 PMC6662593

[108] Colhoun HM McKeigue PM Davey Smith G. Problems of reporting genetic associations with complex outcomes. Lancet 2003;361:865–72. 12642066 10.1016/s0140-6736(03)12715-8

